# A transdisciplinary team approach to scoping reviews: the case of pediatric polypharmacy

**DOI:** 10.1186/s12874-018-0560-4

**Published:** 2018-10-04

**Authors:** Paul M Bakaki, Jennifer Staley, Rujia Liu, Neal Dawson, Negar Golchin, Alexis Horace, Hannah Johnson, Jennifer Waldron, Almut Winterstein, Lawrence C Kleinman, Shari D Bolen

**Affiliations:** 1 Department of Population & Quantitative Health Sciences, School of Medicine, Case Western Reserve University, 10900 Euclid Avenue, Cleveland, OH 44106 USA; 2 Rainbow Babies and Children’s Hospital, University Hospitals, Cleveland, OH USA; 3 Department of Medicine, MetroHealth Medical Center, Cleveland, OH USA; 4 School of Pharmacy, University of Washington, Seattle, WA USA; 5 Department of Clinical Sciences, University of Louisiana at Monroe College of Pharmacy, Monroe, LA USA; 6 School of Medicine, Case Western Reserve University, Cleveland, OH USA; 7 College of Pharmacy, University of Florida, Gainesville, FL USA; 8 The Center for Child Health and Policy at Rainbow, University Hospitals, Cleveland, OH USA; 9 Center for Health Care Research and Policy, Case Western Reserve University at the MetroHealth System, Cleveland, OH USA

**Keywords:** Transdisciplinary research, Team science, Scoping review, Pediatric polypharmacy, Collaborative research

## Abstract

**Background:**

Polypharmacy can be either beneficial or harmful to children. We conducted a scoping review to examine the concept of pediatric polypharmacy: its definition, prevalence, extent and gaps in research. In this manuscript, we report our transdisciplinary scoping review methodology.

**Methods:**

After establishing a transdisciplinary team, we iteratively developed standard operating procedures for the study’s search strategy, inclusion/exclusion criteria, screening, and data extraction. We searched eight bibliographic databases, screened abstracts and full text articles, and extracted data from included studies using standardized forms. We held regular team meetings and performed ongoing internal validity measurements to maintain consistent and quality outputs.

**Results:**

With the aid of EPPI Reviewer collaborative software, our transdisciplinary team of nine members performed dual reviews of 363 included studies after dual screening of 4398 abstracts and 1082 full text articles. We achieved overall agreement of 85% and a kappa coefficient of 0.71 (95% CI 0.68–0.74) while screening full text articles. The screening and review processes required about seven hours per extracted study. The two pharmacists, an epidemiologist, a neurologist, and a librarian on the review team provided internal consultation in these key disciplines. A stakeholder group of 10 members with expertise in evidence synthesis, research implementation, pediatrics, mental health, epilepsy, pharmacoepidemiology, and pharmaceutical outcomes were periodically consulted to further characterize pediatric polypharmacy.

**Conclusions:**

A transdisciplinary approach to scoping reviews, including internal and external consultation, should be considered when addressing complex cross-disciplinary questions.

**Electronic supplementary material:**

The online version of this article (10.1186/s12874-018-0560-4) contains supplementary material, which is available to authorized users.

## Background

Exposure to multiple medications is common among pediatric patients in both inpatient and outpatient settings [1–4]. Using multiple medications can have benefits such as augmentation, tolerability, and efficacy [5–7], or harms such as adverse drug reactions, drug-drug interactions, difficulty adhering to complex regimens, toxicity, overdose, and increased cost [8–12]. Balancing harms and benefits is challenging for prescribers, especially with limited evidence to guide treatment in pediatric patients, and varying definitions and methodologies for pediatric polypharmacy [7, 13, 14]. In the pediatric literature, the term polypharmacy is commonly used when more than one medication is prescribed for a single disease, despite the reality that it is more common to use multiple medications in patients with comorbid diagnoses [15–18]. In this context, we conducted a transdisciplinary scoping review to map the pediatric polypharmacy literature, identify gaps in literature, clarify the definition, and describe the prevalence of pediatric polypharmacy. Transdisciplinary team science is the most integrated form of collaborative research, after interdisciplinary and multidisciplinary approaches [19]. The three terms refer to the involvement of multiple disciplines interacting in different ways while examining aspects of the same larger question or problem. Multidisciplinary is the most basic level of involvement where each discipline independently applies knowledge and approaches, often in a sequential fashion. Interdisciplinary involves concurrent reciprocal interaction among disciplines, necessitating a degree of blurring of disciplinary boundaries. Transdisciplinary transcends disciplinary boundaries to consider the dynamics of systems in a holistic way [20]. To achieve the highest level of collaboration over the broad topic of pediatric polypharmacy, we selected the transdisciplinary team approach for our scoping review.

A scoping review is a form of knowledge synthesis that addresses an exploratory research question aimed at mapping key concepts, types of evidence, and gaps in research related to a defined area or field [21–23]. Like a systematic review, a scoping review uses transparent and reproducible processes to define a research question, search for studies, and synthesize findings. However, a systematic review typically focuses on a well-defined question and aims to provide answers to questions from a relatively narrow range of studies assessed for quality. A scoping study, in contrast, uses the literature to address a broader research question, typically without assessing quality of studies [21, 24, 25]. Scoping reviews seek to reveal patterns that emerge from the literature and are more likely to be hypothesis generating, whereas systematic reviews often are hypothesis testing [26].

We believe that applying a transdisciplinary approach to a scoping review is novel in pediatric literature synthesis. A transdisciplinary team approach is holistic in that team members from different disciplines exchange expertise, knowledge and skills around a complex problem, resulting in integrated insights not easily assigned to a particular discipline [19, 20]. Representatives of different disciplines transcend their separate conceptual, theoretical, and methodological orientations in order to develop a common conceptual framework, in ways that go beyond multidisciplinary and interdisciplinary approaches [27–29]. In multidisciplinary approaches, different disciplines work in parallel or sequentially with the research questions and methods staying within the boundaries of each discipline [20, 28]. Interdisciplinary approaches harmonize links between, typically two, disciplines while mostly maintaining disciplinary identities without a common framework [20]. Success of these collaborative initiatives depends on the extent to which cross disciplinary integrations are actually achieved by research teams [30].

A common feature of scoping studies has been the need to engage researchers from a wide range of academic disciplines [31]. Additionally, authors may find it appropriate to search multiple sources of literature— quantitative, qualitative, text, opinion pieces, or summaries [32–34].

Multidisciplinary approaches have been widely used in scoping studies [35, 36]. A multidisciplinary team provides the required specialist knowledge to map a subject that is not necessarily always found in one field [22, 31]. This approach can, however, create problems as researchers from very different theoretical perspectives often have difficulty in working together [31]. Because of its intentional integrational nature, the transdisciplinary approach may be better suited to handle the broad research questions and types of literature of scoping studies [37]. Similar to the transdisciplinary approach, an integrated knowledge translation approach [38] is a collaborative research process whereby researchers and knowledge users work together from developing the question through designing, completing the literature search, analyzing and interpreting the data and disseminating the results [26].

While multiple research teams have conducted narrative, scoping, and systematic reviews on polypharmacy among adults and the elderly [39–42], we were unable to find transdisciplinary teams that conducted systematic or scoping reviews of studies involving pediatric polypharmacy. In this manuscript, we report the methods of our transdisciplinary scoping review of pediatric polypharmacy. To facilitate potential replication by others, we also describe the key roles of our transdisciplinary team members.

## Methods

### Study design and methodology

We used the methodological framework for scoping reviews proposed by Arksey and O’Malley, and enhanced by others [21–23, 32, 43–45]. We specifically adopted Levac and colleagues’ modifications including the steps outlined below [44]. Our detailed protocol is available from the corresponding author upon request.

1. Articulate the research question in relation to the purpose and rationale of the study.
2. Identify relevant studies while considering human and financial resources, breadth, and comprehensiveness.
3. Select studies using an iteratively developed search strategy, and abstract and full text inclusion and exclusion criteria, applied independently by two reviewers.
4. Chart the data by two reviewers using a collectively and interactively developed extraction form.
5. Collate, summarize, and report the results in relation to study purpose, and implications for policy, practice, or research
6. Conduct ongoing engagement and consultation of experts to further understand the concept of pediatric polypharmacy

### Transdisciplinary team approach

Our transdisciplinary team included core disciplines of pediatrics, pharmacy, evidence synthesis, epidemiology, and library and information science. We developed three sub-teams: the implementation team, the protocol team, and the project stakeholders or consultants. The implementation team was comprised of ten members who developed the protocol and standard operating procedures (SOPs), collected data and drafted the manuscripts. The protocol team - a group of three experts in internal medicine, research implementation, pharmaceutical outcomes research, pediatrics, health services research and policy - oversaw the development and implementation of the research by mentoring and advising the implementation team. Project stakeholders/consultants included experts in content areas critical to our research, including mental health, childhood complex chronic disease conditions, pediatrics, epilepsy, pharmacoepidemiology, and scoping research methodology. We consulted with members of this group during protocol development, data interpretation, and reporting.

**Fig. 1:**
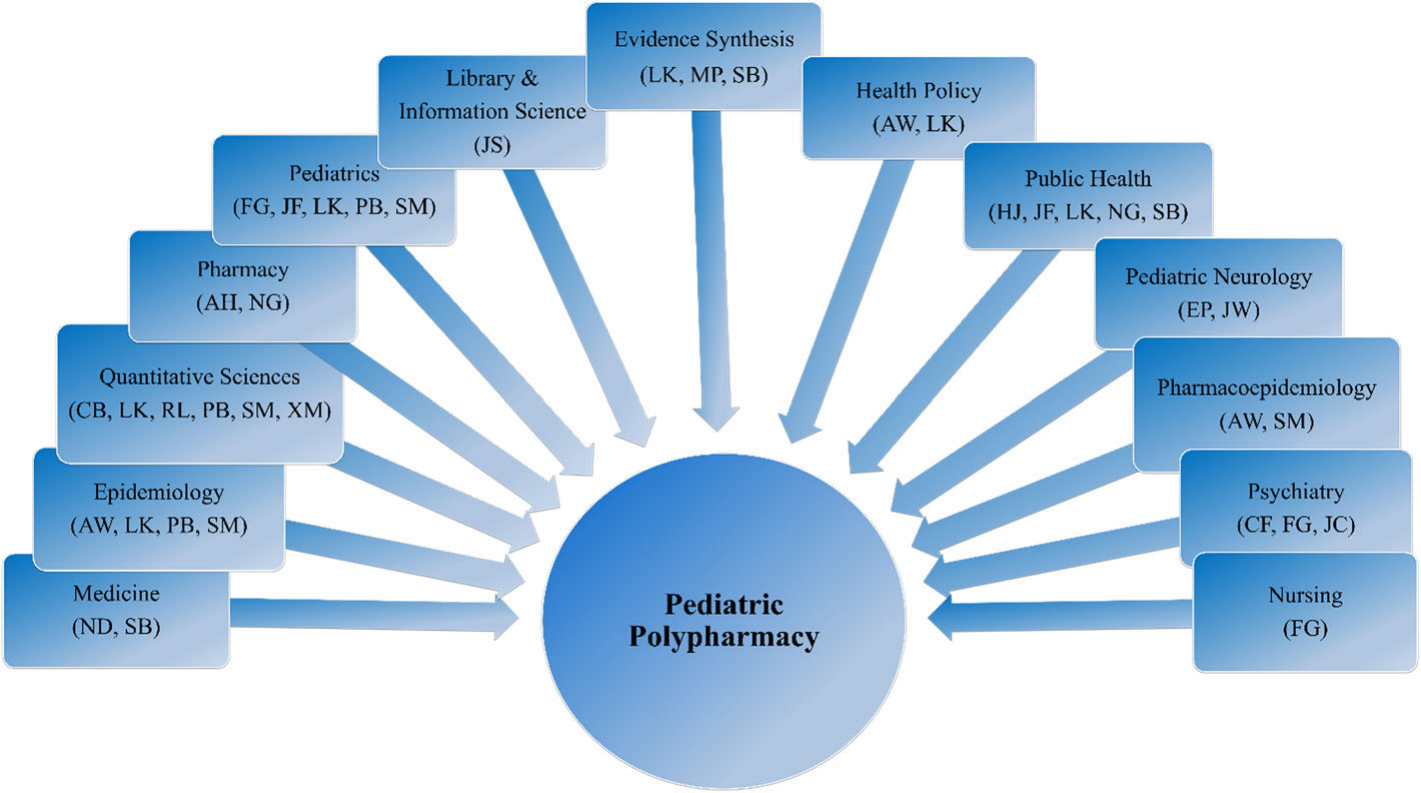
The Pediatric Polypharmacy *Transdiscipline* Schema**.** Implementation Team: Alexis Horace (AH), Courtney Baker (CB), Hannah Johnson (HJ), Jennifer Staley (JS), Jennifer Waldron (JW), Negar Golchin (NG), Paul Bakaki (PB), Rujia Liu (RL), Shari Bolen (SB), Xuan Ma (XM). Protocol Team: Almut Winterstein (AW), Larry Kleinman (LK), Neal Dawson (ND). Stakeholders/Consultants: Cynthia Fontanella (CF), Elia Pestana (EP), Faye Gary (FG), James Feinstein (JF), Joseph Calabrese (JC), Mai Pham (MF), Sharon Meropol (SM)

We identified and recruited members to our team locally from three large hospital systems, a medical school, and a nursing school, and nationally, including authors of seminal manuscripts in the field. Graduate research assistants brought further relevant disciplinary expertise, including biostatistics and public health, into the team. We illustrate our transdisciplinary team in Fig. 1. The overlapping boxes and fading color depict the merging and fading of the individual disciplines into a new distinct transdisciplinary product. A team leader coordinated the activities of our transdisciplinary team.

The implementation team met weekly, in person and via teleconference, while the broader team communication was primarily electronic. Table 1 summarizes the activities of our transdisciplinary implementation team, designed to foster communication and cooperation, and to ensure quality research outputs. We conducted training sessions for the implementation team at critical stages of the study, including when the team was newly created, when new members joined the team, and before the beginning of the pilot and implementation phases. We used weekly meetings to discuss challenging studies and share information about progress, interrater agreement, weekly targets, and quality improvement. The epidemiologist, librarian, pharmacists, pediatric neurologist, and evidence synthesis expert on the implementation team provided disciplinary perspectives during training, SOP development, and review of studies. For example, the epidemiologist helped to incorporate principles of study design; pharmacists guided the classification of medications; clinicians clarified clinical meaning; and the librarian guided the team towards best practices in literature search. Each played an integral role in teaching the team respective disciplines to help in the execution of the project. These experts on our implementation team also screened studies and extracted data (Additional file 1), requiring them to learn and apply skills from other disciplines. For example, the librarian participated in conceptualizing the project, screening and extracting data from studies, and drafting abstracts and manuscripts in addition to searching for studies.

**Table 1:** Activities and Procedures of the Pediatric Polypharmacy Transdisciplinary Team

Item	Purpose	Activities
Weekly Meeting	Training	Share experiences, discuss difficult studies, refine Standard Operating Procedures (SOPs), and review administrative issues
Appreciative Inquiry [46]	To optimize collaboration and team unity	Answer questionnaire about strengths and expectations
“Letter to Mum” I	To appreciate scoping review as a method and improve the ability to communicate with people of diverse backgrounds about what we do	Write a letter to explain in a straightforward and succinct way what a scoping review study is and why it is important
“Letter to Mum” II	To learn how to conduct and report scoping study results to people of diverse backgrounds	Write a letter to explain how to conduct and report a scoping study in clear and easy to understand language
Dual Review	To enhance accuracy of screening and enhance cooperation	Independently screen and compare results for titles, abstracts, and full text; discuss disagreements
Weekly E-mail	To enhance efficiency, standardization, and clarity	Communicate with team about weekly schedule, progress, and clarify ambiguity
Google Drive	To share and edit files efficiently	Share documents and files, collaborate on documents, and download files
Meet Team Leader	To address person-specific needs and questions, and give bilateral feedback	Two weekly individual meetings with team leader
Training Sessions	To achieve efficiency, accuracy and standardization	New member or group training at team entry and before each major study phase
SOPs	Training and standardization	Iteratively write and edit SOPs

The incorporation of internal content experts was efficient and informative when faced with the many technical, pharmacological, and clinical data challenges in the literature. Even so, medication classification remained challenging because of the existence of multiple classification systems and the presence of apparent misclassifications in the literature. The ready availability of pharmacists on the team made it possible to complete the work despite this lack of clarity.

The team made use of a shared Google Drive account to create and/or store research documents including the protocol, responses to an appreciative inquiry questionnaire [46], “letters to Mum” (described in Table 1), seminal methodological manuscripts, meeting minutes, SOPs, draft abstracts, and manuscripts. We used the tool “letter to Mum” to convey the need to communicate our research to lay people, referring to one of our team member’s mum who is unschooled.

### Protocol

The team leader (PB) conceptualized the research questions and drafted the research protocol in consultation with the evidence synthesis expert (SB) and librarian (JS). The rest of the team, including our stakeholders, reviewed, edited, and approved the protocol before implementation. This iterative process ensured that the experts generated transdisciplinary research questions and approaches. We made a few modifications to the protocol during implementation to refine our inclusion and exclusion criteria in keeping with scoping review methodology. For example, while our search strategy specified children, we decided to retain any resulting studies that included a few young adults up to age 25 when they predominantly studied children below age 19 and were conducted in pediatric facilities. The team leader drafted the initial screening and data extraction forms which, with input from the implementation team, underwent considerable changes during the pilot and implementation phases of the study. This process aligns with the iterative nature of scoping reviews [21]. These modifications were valuable as they were informed by new insights, discoveries, prior omissions, and the need for clarification discovered during study screening and data extraction. As a function of EPPI Reviewer-4 software [47], our screening and data extraction forms were able to accommodate reference information explaining the meaning of each field and how it should be completed. These SOPs were developed iteratively [21] by our core implementation team. They were also available to team members as stand-alone electronic copies in our shared Google Drive account for reference. The SOPs, screening, and data extraction forms were piloted on 100 studies. Twenty-one of these studies were included in the final data.

### Eligibility

We sought to review original observational studies written in English that assessed polypharmacy in children 18 years of age or younger. Studies must have assessed polypharmacy as an aim, outcome measure, main predictor of outcome, or a covariate. We did not impose any geographical or publication year limits. Neither did we consider study quality, as is common in scoping reviews. We excluded case reports; non-English or non-original studies (reviews, opinion pieces, letters, or abstracts); studies exclusively conducted in adult subjects; those not about polypharmacy; or those that assessed polypharmacy experimentally, prenatally, or during breastfeeding. We excluded experimental research because our study aims involved gaining understanding regarding the practice patterns, definition, and prevalence of pediatric polypharmacy, which cannot be addressed by experimental studies.

### Search strategy

**Fig. 2:**
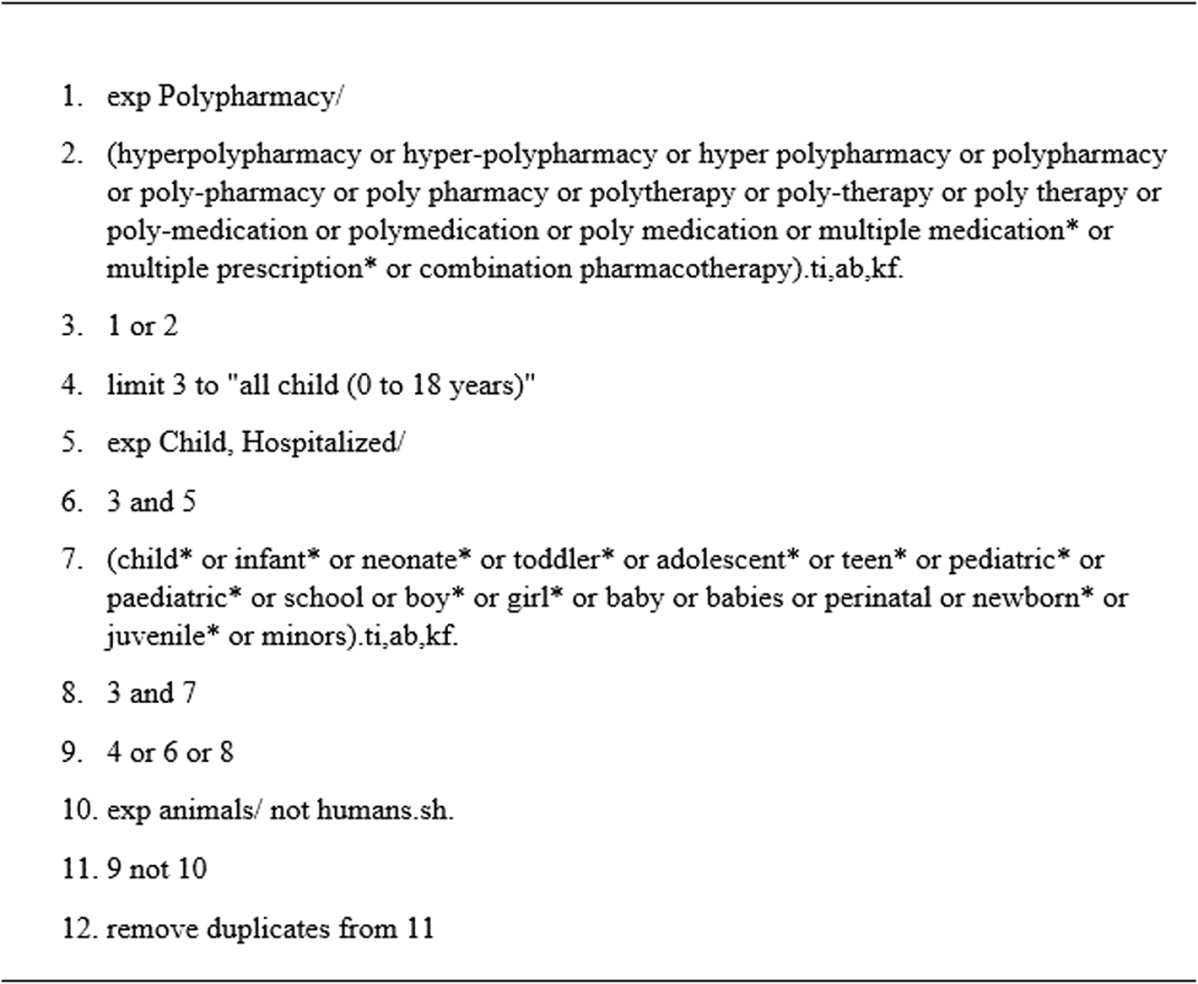
Medline Search Strategy

Our librarian developed a search strategy in consultation with the team leader and evidence synthesis expert. The final MEDLINE search strategy is shown in Fig. 2. First, we searched the following databases from inception to October 2016 using controlled vocabulary and free text terms for the concepts of polypharmacy and children: Ovid Medline, PubMed, EMBASE, CINAHL, Ovid PsycINFO, Cochrane CENTRAL, ProQuest Dissertations & Theses A&I, and the Web of Science Core Collection. We then updated the database search in July 2017. Finally, we hand searched a random sample of 30 studies (10% of included studies from the original search), as well as six relevant review articles to find any studies our database search might have missed. The six review articles were purposively selected by two reviewers, out of 15 identified by the whole review team, prioritizing medications studied (all medications, antipsychotics, antiepileptic), age groups (neonates, older children), and outcomes (e.g. adverse drug reactions).

### Study selection

Once we acquired relevant titles and abstracts, two independent reviewers screened titles and abstracts together using a standardized abstract screening form. Likewise, pairs of independent reviewers screened full text articles using an article screening form. Reviewer pairs met in-person or by telephone to reconcile differences after independently reviewing their studies. Conflicts were resolved by the larger implementation team.

### Data extraction

We iteratively designed a comprehensive data extraction form to collect information pertaining to study and population characteristics, study measures, definition of polypharmacy, diseases and medications evaluated. Sections of the extraction form were initially drafted by members of the relevant disciplines (e.g. medications by pharmacists, disease conditions by pediatricians, and study measures by epidemiologists) then they were discussed and revised by the transdisciplinary team. Data extraction was conducted through two sequential reviews. Our detailed data extraction form is provided in Additional file 2.

We timed both screening and data extraction in order to distribute studies according to each reviewer’s availability and review speed. In addition to computing individual average duration, we computed group average duration of screening and data extraction in order to establish an objective timeline of study activities. This tracking did not take into account the immense amount of time spent on project administration, database searches, article retrieval, data cleaning, and processing.

### Data synthesis

We conducted both qualitative and quantitative synthesis of the data. We performed a qualitative synthesis on the definitions of polypharmacy that assessed text definitions for the presence of components such as number of medications, overlap period of medications, class or drug level, medication or class name, and limitation to one or two medications. Additionally, we conducted descriptive statistics of individual variables and cross-tabulated variables to examine relationships. A pharmacist, an epidemiologist, and a library and information scientist conducted the qualitative synthesis in pairs and among all of them in case of disagreements. The quantitative health scientists (epidemiologist and statisticians) performed the descriptive statistics and shared preliminary results with both the review team and consultants for input before generating the final results.

### Software

We used Clarivate Analytics EndNote (X7) to find studies and remove duplicates. We then imported the titles and abstracts into EPPI-Reviewer 4 (EPPI) [47]. We used EPPI for data management and collaborative review. It aided us in randomly assigning studies to groups, creating screening and data extraction forms, screening studies and reconciling differences, data cleaning, data synthesis, and report generation. We used SAS and Microsoft Excel to compute Kappa statistics, process, and analyze data downloaded from EPPI.

### Quality control

Implementing quality control measures such as training, independent paired reviews and the use of standardized data collection forms reinforced the reliability of our study procedures. We screened titles and abstracts in batches of 120 randomly assigned studies and extracted data from 20 randomly assigned full text studies per pair of reviewers. The team leader purposively assigned these groups to varying review pairs to maximize interactions between team members based on availability, experience, and academic discipline. We measured inter-rater reliability using percent agreement and Kappa statistic (*k*) [48]. Percent agreement for each group of studies was immediately available to the reviewer pair. The team leader also generated cumulative percent agreement on a weekly basis to provide insight on screening uniformity. We discussed strategies for improving agreement during our weekly team meetings. Kappa statistic was computed for a 30% sample of all titles and abstracts screened as well as for all full text studies screened.

## Results

### Studies reviewed

Of 8651 citations, we screened 4398 studies. A total of 363 studies remained for data extraction and synthesis. A full list of the 363 included studies is provided in Additional file 3.

### Time investment

The pilot phase lasted 3 months, implementation lasted 8 months, data cleaning and processing lasted 2 months. Individual time commitment varied among team members (Additional file 1). On average, it took 7 min to screen a study on title and abstract, 13 min to screen on full text, 76 min to conduct a primary data extraction, 52 min to verify the extraction, and 30 min to address queries raised through the verification and data cleaning process. Dividing the total time spent on the reviews by the 363 included studies revealed that it took 7.3 h to produce a record in our analytic database. We required reviewers to time only their uninterrupted reviews which were 61% (220/363) of included studies.

### Inter-rater concordance

The Kappa coefficients (*k*) ranged from fair to excellent (0.41–0.8) [48] for all screening on title and abstract or full text, with/without reason for exclusion (Additional file 4). Inter-reviewer agreement was highest when reviewers were similar to one another, for example, between pharmacists or between graduate assistants. The agreement was lowest between dissimilar reviewers, especially during the early phase of the implementation period. Pharmacists tended to go “deeper” when extracting information related to medicines and physicians went “deeper” when extracting information about disease conditions while non clinicians were uniform while extracting information. Inter-reviewer agreement increased as the study progressed, irrespective of reviewer similarity, mainly because we tasked specialists to simplify professional language while training others and writing SOPs.

## Discussion

In this project, we established a transdisciplinary team of experts that conducted a large scoping review in order to map the pediatric polypharmacy literature, identify research gaps, clarify the definition of pediatric polypharmacy, and describe the prevalence of pediatric polypharmacy. In addition to reviewing studies, the team also provided internal consultation to one another throughout the project cycle.

### Study strengths

In the past, some scoping reviews have been conducted by multidisciplinary [35] and inter-professional [23] teams while other large scoping reviews have depended on a few individuals to conduct the review [29, 49]. What distinguishes our project from many others is integration of the transdisciplinary approach at every stage of the scoping review [29]. Five out of ten members of our implementation team participated in acquiring studies, nine reviewed studies, and eight were involved in drafting abstracts and manuscripts. Achieving this level of participation required learning about other disciplines and teaching one’s own discipline to each other. We illustrate this integrated effort through the description of activities of our health science librarian, a discipline that has been found lacking among authors of scoping reviews [50]. The librarian was engaged during the whole scoping review cycle: conceptualizing the research question; developing database search strategies; retrieving studies, screening studies; extracting data; training team members; reviewing SOPs; and writing abstracts, posters, and manuscripts. Similar experiences were mirrored among members of the other core disciplines including pharmacists, clinicians, and research methodologists.

With a librarian, epidemiologist, two pharmacists, and a clinician reviewing studies, we had internal consultation in our core disciplines. Moreover, we had a knowledge synthesis expert attend our weekly meetings to address pressing issues in real time. The transdisciplinary approach where we constantly worked on establishing a common strategy enabled us to perform internal consultation at least as rigorously as the inter-professional approach used by Daudt and colleagues [23]. The episodic engagement of the project stakeholders provided a second layer of consultation.

### Time requirement

As other authors of scoping reviews have argued previously, a scoping review is not a rapid review [23, 50] as it was initially defined [21]. In our situation, it took thirteen months to complete the project. Lack of published guidance on detailed time frames made it difficult for us to project how much time each stage would take. The detailed extraction form partially explains why it took our group more than 2 h for each study. However, it is not likely that a shorter extraction form would have significantly reduced the amount of time needed to extract a study. Additionally, a shorter form would not have adequately met the needs of our broad research questions. One potential option for time saving could have included utilizing the machine learning capability available in EPPI Reviewer-4 (EPPI) software for screening on title and abstract. By our estimates, automated screening would have reduced our review time by about 800 h or 30% of the total time. This approach, however, would still require manually screening about 1000 random titles and abstracts from which EPPI would learn in order to screen the rest of the studies. In any case, we hope that future researchers planning scoping reviews similar to ours can use the 7 h estimate for an extracted study to plan their own projects.

### Limitations

Our scoping review project had some limitations. As expected in a large team like ours, member involvement varied with time due to competing engagements and the unpredictable time requirements of our study. The team leader ensured that each person was assigned work proportional to their availability during each phase of the study. Setting short-term targets, typically of one to 2 weeks, helped to ensure continuity. Converging our team’s diverse training, experiences, and backgrounds around the concept of pediatric polypharmacy required more time, discussions, and meetings among team members.

Whereas we achieved kappa coefficients in the moderate to substantial range of 0.41–0.80 [48], the coefficients for screening on title and abstract were lower than those for screening on full text. This may be explained by the difference in inclusion proportions at title and abstract screening of 25% and at full text screening of 30%. The opportunity for chance agreement to increase (and for kappa coefficients to decrease) is enhanced with the deviation of inclusion proportion away from 50% [51]. Overall, both percent agreement and kappa coefficients increased as the study progressed, implying mastery of screening guidelines with time and practice. The high agreement we observed between reviewers of the same discipline seems to imply similarity of background knowledge rather than protocol-based decisions. Minimizing the paring of reviewers with similar disciplinary backgrounds and changing reviewer pairs of study batches between the screening phases addressed this problem.

Excluding experimental and non-primary observational studies such as review studies may have led to exclusion of some definitions of pediatric polypharmacy, affecting one of the primary aims of this study.

## Conclusions

In conclusion, we completed a scoping review as a transdisciplinary team. Our team efforts have enabled us to implement a project of considerable magnitude, as well as benefit from all team members’ skills and efforts. Our transdisciplinary review team provided inbuilt expert consultation which complemented that from external stakeholders. We found holding regular meetings, producing SOPs, and continually assessing internal validity to be helpful.

We recommend that researchers conducting large scoping reviews consider using a transdisciplinary approach to elevate the breadth and focus of the questions; enhance the scoping review decisions and iterations; and enrich review conclusions. We hope the detailed description provided in this paper will help others conducting future scoping reviews when designing their studies and planning for necessary resource requirements.

## Additional files

Additional file 1: Distribution of Studies Reviewed and Time Spent by the Pediatric Polypharmacy Scoping Review Implementation Team. Reviewer work load and amount of time spent on abstract screening, full text screening, and data extraction. (DOCX 17 kb)

Additional file 2: Data Extraction Form. All the questions used for extracting data. (DOCX 24 kb)

Additional file 3: Included Studies. List of first author, year of publication, and title of all included studies. (DOCX 54 kb)

Additional file 4: Inter-rater Concordance Measures for Screening on Title & Abstract, and Full Text**.** Details of percent agreement and kappa statistics for screening title & abstracts as well as full texts. (DOCX 19 kb)

## Abbreviations

EPPI: EPPI-Reviewer 4
SAS: Statistical analysis system
SOP: Standard operating procedures
