# Supplementary material for: A transdisciplinary team approach to scoping reviews: the case of pediatric polypharmacy
Source: BMC Med Res Methodol. 2018 Oct 4;18:102. doi: 10.1186/s12874-018-0560-4 (PMC6172739; doi:10.1186/s12874-018-0560-4)
Supplement: Supplementary file 1 — Distribution of Studies Reviewed and Time Spent by the Pediatric Polypharmacy Scoping Review Implementation Team. Reviewer work load and amount of time spent on abstract screening, full text screening, and data extraction. (DOCX 17 kb) [file 12874_2018_560_MOESM1_ESM.docx]

**Additional File 1: Distribution of Studies Reviewed and Time Spent by the Pediatric Polypharmacy Scoping Review Implementation Team**

| **Reviewer** | **Position** | **Titles & Abstracts** | **Full Text Reviews** | **Extractions (Primary)** | **Extractions (Secondary)** | **Time (Hours)** |
| --- | --- | --- | --- | --- | --- | --- |
| **1** | Epidemiologist (Team Leader) | 1,645 | 320 | 73 | 69 | **502** |
| **2** | Librarian | 1,002 | 154 | 6 | 14 | **173** |
| **3** | Clinical Fellow (Pediatric Neurology) | 794 | 145 | 2 | 8 | **135** |
| **4** | Pharmacist 1 | 1,292 | 31 | 15 | 6 | **189** |
| **5** | Pharmacist 2 | 1,086 | 121 | 23 | 12 | **204** |
| **6** | Research Assistant 1 (Anthropology/Public Health) | 2,660 | 588 | 20 | 71 | **535** |
| **7** | Research Assistant 2 (Biostatisics) | 237 | 285 | 86 | 74 | **311** |
| **8** | Research Assistant 3 (Biostatistics/Engineering) | 302 | 255 | 80 | 47 | **271** |
| **9** | Research Assistant 4 (Business Engineering/Biostatisics) | 171 | 273 | 87 | 94 | **329** |
| **Total** |  | **9,189** | **2,172** | **397** | **397** | **2,649** |

1. On average, it took 7 minutes to screen a study on title and abstract, 13 minutes to screen on full text, 76 minutes to conduct a primary data extraction, 52 minutes to verify the extraction, and 30 minutes to address querries
2. 363 out of 397 extracted studies were included in the final data analysis, 34 were excluded at extraction phase
