# Supplementary material for: A transdisciplinary team approach to scoping reviews: the case of pediatric polypharmacy
Source: BMC Med Res Methodol. 2018 Oct 4;18:102. doi: 10.1186/s12874-018-0560-4 (PMC6172739; doi:10.1186/s12874-018-0560-4)
Supplement: Supplementary file 2 — Data Extraction Form. All the questions used for extracting data. (DOCX 24 kb) [file 12874_2018_560_MOESM2_ESM.docx]

**Additional File 2: Data extraction Form**

- Linked items
- HOW CAN THE STUDY BE IDENTIFIED?
  - #001Study Phase
    - Pilot
    - Implementation
    - Update
    - HandSearch
  - #002Status of report
    - Published
    - InPress
    - Unpublished
  - #003Year of report
    - Upto1975
    - 1976to1980
    - 1981to1985
    - 1986to1990
    - 1991to1995
    - 1996to2000
    - 2001to2005
    - 2006to2010
    - 2011+
  - #004Years study was conducted
    - NotReported(WRITE'9999')
    - StartYear(WRITE)
    - EndYear(WRITE)
  - #005Country where study was implemented (may check >1)
    - NotReported
    - Australia
    - Brazil
    - Canada
    - China
    - Ethiopia
    - Finland
    - France
    - Germany
    - Guyana
    - Hong Kong
    - India
    - Indonesia
    - Ireland
    - Israel
    - Italy
    - Japan
    - Jordan
    - Lebanon
    - Malaysia
    - Nepal
    - Nigeria
    - Norway
    - Netherlands
    - Poland
    - South Africa
    - South Korea
    - Spain
    - Sweden
    - Switzerland
    - Taiwan
    - Turkey
    - UK
    - USA
    - Other(NEXT QST)
  - #006Other Country1 (WRITE)
  - #007Other Country2 (WRITE)
  - #008Other Country3 (WRITE)
  - #009What type of study was it?
    - Original study
    - Review Study
    - Dissertation
    - Conference proceeding
    - Institutional report
    - Other (NEXT QST)
  - #010Other Study Type (WRITE)
  - #011(a) What was the study design?
    - Not reported
    - Qualitative study
    - Cross-sectional study
    - Retrospective cohort study
    - Prospective cohort study
    - Clinical (experimental) trial
    - Case control
    - Other(NEXT QST)
  - #011(b)Other Study Design (WRITE)
  - #012Data source(s) (may check >1)
    - Not reported
    - Chart review
    - Electronic health records
    - Insurance claims
    - Primary data collection
    - (Drug) registry
    - School records
    - Social media
    - Literature review
    - Lab
    - Other(ANSWER NEXT QST)
  - #013Other data source1 (WRITE in info box)
  - #014Other data source2 (WRITE in info box)
- DESCRIPTION OF STUDY SAMPLE
  - #015Children only study?
    - ChildrenOnly
    - Children&AdultStratifiied
    - Children&AdultsNotStratified
  - #016Number child participants-Categorized
    - NotReported
    - <50
    - 50-99
    - 100-499
    - 500-999
    - 1000+
  - #017a Number of participants (may check >1)
    - Not reported (WRITE '99' in info box)
    - Number of children (WRITE in info box)
    - Total number of participants (WRITE in info box)
  - #017b Population (characteristics) (WRITE in info box)
- AGE DISTRIBUTION
  - #018Mean Age and Range
    - Not reported (WRITE '00' in info box)
    - Mean age (WRITE in info box)
    - Standard deviation (WRITE in info box)
    - Age minimum (WRITE in info box)
    - Age maximum (WRITE in info box)
    - Age range (WRITE in info box)
    - Median age (WRITE in info box)
    - Quartile1 (WRITE in info box)
    - Quartile3 (WRITE in info box)
  - #019Age groups (may check >1)
    - NotReported
    - 0AgeCat
    - 1-4AgeCat
    - 5-12AgeCat
    - 13-17AgeCat
    - 18-21AgeCat
- REPORTED AGE GROUPS
  - #020Age Categories Reported?
    - Yes
    - No
  - #021Reported age group1
    - Not reported (WRITE '99' in info box)
    - Lower limit (WRITE in info box)
    - Upper limit (WRITE in info box)
    - Percent (WRITE in info box)
  - #022Reported age group2
    - Not reported (WRITE '99' in info box)
    - Lower limit (WRITE in info box)
    - Upper limit (WRITE in info box)
    - Percent (WRITE in info box)
  - #023Reported age group3
    - Not reported (WRITE '99' in info box)
    - Lower limit (WRITE in info box)
    - Upper limit (WRITE in info box)
    - Percent (WRITE in info box)
  - #024Reported age group4
    - Not reported (WRITE '99' in info box)
    - Lower limit (WRITE in info box)
    - Upper limit (WRITE in info box)
    - Percent (WRITE in info box)
  - #025Reported age group5
    - Not reported (WRITE '99' in info box)
    - Lower limit (WRITE in info box)
    - Upper limit (WRITE in info box)
    - Percent (WRITE in info box)
  - #026Reported age group6
    - Not reported (WRITE '99' in info box)
    - Lower limit (WRITE in info box)
    - Upper limit (WRITE in info box)
    - Percent (WRITE in info box)
- SEX
  - #027SEX Reported?
    - Yes
    - No
  - #028SEX Male
    - Not reported (WRITE '99' in info box)
    - Number male (WRITE in info box)
    - Percent male (WRITE in info box)
- HEALTH INSURANCE
  - #029Health Insurance Reported
    - Yes
    - No
  - #030Public insurance (e.g. Medicaid, government)
    - NotReported(WRITE'99')
    - Number(WRITE)
    - Percent(WRITE)
  - #031Private insurance
    - NotReported(WRITE'99')
    - Number(WRITE)
    - Percent(WRITE)
  - #032Uninsured
    - NotReported(WRITE'99')
    - Number(WRITE)
    - Percent(WRITE)
  - #033Other insurance1
    - NotReported(WRITE'99')
    - Category(WRITE)
    - Number(WRITE)
    - Percent(WRITE)
  - #034Other insurance2
    - NotReported(WRITE'99')
    - Category(WRITE)
    - Number(WRITE)
    - Percent(WRITE)
- RACE
  - #035RACE Reported?
    - Yes
    - No
  - #036White non-Hispanic
    - NotReported(WRITE'99')
    - Number(WRITE)
    - Percent(WRITE)
  - #037Black non-Hispanic
    - NotReported(WRITE'99')
    - Number(WRITE)
    - Percent(WRITE)
  - #038Hispanic or Latino
    - NotReported(WRITE'99')
    - Number(WRITE)
    - Percent(WRITE)
  - #039Asian or Asian American
    - NotReported(WRITE'99')
    - Number(WRITE)
    - Percent(WRITE)
  - #040Other race1
    - NotReported(WRITE'99')
    - Race(WRITE)
    - Number(WRITE)
    - Percent(WRITE)
  - #041Other race2
    - NotReported(WRITE'99')
    - Race(WRITE)
    - Number(WRITE)
    - Percent(WRITE)
  - #042Other race3
    - NotReported(WRITE'99')
    - Race(WRITE)
    - Number(WRITE)
    - Percent(WRITE)
- STUDY AIMS AND MEASURES
  - #043Was polypharmacy the primary study aim?
    - Yes
    - No
  - #044Aim(s)(MayCheck>1)
    - NotRereported(WRITE)
    - Aim1(PASTE)
    - Aaim2(PASTE)
    - Aim3(PASTE)
    - Aim4(PASTE)
  - #045What terms describe concurrent use of multiple medications (may check >1)?
    - Not reported
    - Add-on
    - Adjunctive
    - Augmentation
    - (Average) number of meds
    - Combination pharmacotherapy
    - Comedication
    - Concomitant meds
    - Concurrent medication
    - Coprescription
    - Dual therapy
    - Multiple medications
    - Multiple prescription
    - Polypharmacy
    - Polytherapy
    - Simultaneous
    - Other (ANSWER NEXT QST)
  - #046Other poly term1 (WRITE in info box)
  - #047Other poly term2 (WRITE in info box)
  - #048Other poly term3 (WRITE in info box)
- OUTCOMES OF INTEREST
  - #049Was polypharmacy the primary outcome?
    - Yes
    - No
  - #050What was the primary outcome of interest?
    - NotReported
    - Polypharmacy
    - ADRs
    - DDIs
    - PoorAdherence
    - ERVisit
    - Hospitalization
    - Death
    - Prognostic
    - Pharmacokinetic
    - Medication Use
    - Cost
    - Diagnostics
    - Other(NEXT)
  - #051Other primary outcome1 (WRITE in info box)
  - #052Other primary outcome2 (WRITE in info box)
  - #053What were the secondary outcomes (may check >1)
    - NotReported
    - Polypharmacy
    - ADRs
    - DDIs
    - PoorAdherence
    - ERVisit
    - Hospitalization
    - Death
    - Prognostic
    - Pharmacokinetic
    - Medication Use
    - Cost
    - Diagnostics
    - Other(NEXT)
  - #054Other secondary outcome1 (WRITE in info box)?
  - #055Other secondary outcome2 (WRITE in info box)?
- MAIN PREDICTORS
  - #056Was polypharmacy the MAIN predictor?
    - Yes
    - No
  - #057MAIN predictor(s) of interest (may check >1)
    - NotReported
    - Polypharmacy
    - Comorbidity
    - DiseaseSeverity
    - ERVisit/IP/OP
    - Pharmacokineitc
    - Treatment
    - Health System
    - Behavioral (co-morbidity)
    - Insurance
    - Age
    - Sex
    - Family/Social/Economic History
    - ADR
    - Other(NEXT)
  - #058Other main predictor1 (write in info box)?
  - #059Other main predictor2 (write in info box)?
  - #060Other main predictor3 (write in info box)?
- OTHER COVARIATES
  - #061What were the other covariates (may check >1)?
    - Not reported
    - Polypharmacy
    - Comorbidity/Pathology
    - Disease severity
    - ER visit/IP/OP visits
    - Age
    - Sex
    - Race/ethnicity
    - Insurance status
    - Socio-economic status
    - weight/height/BMI/HC/G&Developmet
    - Health System
    - Treatment
    - ADR
    - Pharmacokinetic
    - Location
    - Family history
    - Other (WRITE IN NEXT QST)
  - #062Other covariate1 (WRITE in info box)?
  - #063Other covariate2 (WRITE in info box)?
  - #064Other covariate3 (WRITE in info box)?
- DEFINITION OF POLYPHARMACY
  - #065Was polypharmacy defined?
    - YesExplicitly
    - YesImplicitly
    - No
  - #066What was the EXPLICIT definition of polypharmacy (may check >1)?
    - No (explicit) definition (WRITE 'none' in info box)
    - Definition of polypharmacy1 (PASTE in info box)
    - Definition of polypharmacy2 (PASTE in info box)
    - Definition of polypharmacy3 (PASTE in info box)
  - #067What was the source of definition of polypharmacy?
    - No(explicit)Definition
    - OriginalDefinition
    - CitedDefinition(NEXT-QST)
  - #068Cited definition (PASTE citation in info box)
- HEALTHCARE SETTING
  - #069(Healthcare) setting where polypharmacy was assessed (may check >1)
    - Not reported
    - Outpatient
    - Inpatient
    - Pharmacy
    - School
    - Insurance company
    - Foster care/group home
    - Population level
    - Other (ANSWER NEXT QST)
  - #070Other healthcare setting (WRITE in info box)
- PREVALENCE OF POLYPHARMACY
  - #071What was the mean number of meds?
    - Not reported (WRITE '99' in info box)
    - Mean # of meds (WRITE in info box)
    - Median # of meds (WRITE in info box)
  - #072Was prevalence of polypharmacy estimated?
    - NotReported
    - Yes
    - No
  - #073Denominator in computing prevalence
    - NotReported
    - NumberWithMono&Polytherapy
    - NumberWithMono&Polytherapy&NoMeds
    - Other(NEXT-QST)
  - #074Other denominator (WRITE in info box)
  - #075Overall prevalence
    - NotReported(WRITE'999')
    - Numerator(WRITE)
    - Denominator(WRITE)
    - Percent(WRITE)
  - #076Categorical prevalence1
    - Not reported (WRITE '999' in info box)
    - Category (WRITE in info box)
    - Numerator (WRITE in info box)
    - Denominator (WRITE in info box)
    - Percent (WRITE in info box)
  - #077Categorical prevalence2
    - Not reported (WRITE '999' in info box)
    - Category (WRITE in info box)
    - Numerator (WRITE in info box)
    - Denominator (WRITE in info box)
    - Percent (WRITE in info box)
  - #078Categorical prevalence3
    - Not reported (WRITE '999' in info box)
    - Category (WRITE in info box)
    - Numerator (WRITE in info box)
    - Denominator (WRITE in info box)
    - Percent (WRITE in info box)
- MEDICATION OVERLAP
  - #079aWas polypharmacy overlapping or sequential (may check >1)?
    - Overlapping
    - Sequential
  - #079bSequential Period
    - NotReported
    - Overlap
    - HospitalizationPeriod
    - 2-14days
    - 15-30days
    - 31-60days
    - 61-120days
    - 121-180days
    - 181-365days
    - 1-2years
    - >2-5years
    - >5years
    - Other(WRITE in infobox)
  - #080Duration of medication overlap was reported as (may check >1):
    - Not reported
    - Sequential
    - >=1 day
    - >7 days
    - >14 days
    - > 30 days
    - >60 days
    - >90 days
    - >=180 days
    - >=365 days
    - Other (ANSWER NEXT QST)
  - #081Other medication overlap (WRITE in info box)
  - #082Count of overlapping meds was reported as (may check >1):
    - Not reported
    - >= 2 meds
    - >= 3 meds
    - >=4 meds
    - >= 5 meds
    - >= 10 meds
    - Other (ANSWER NEXT QST)
  - #083Other medication count (WRITE) in info box)
  - #084Was polypharmacy classified?
    - NotReported
    - Yes
    - No
  - #085(a) How was polypharmacy classified/characterized/described/qualified (check more than 1)?
    - Not reported (WRITE 'none')
    - Short term (ANSWER CUT OFF Q)
    - Long term (ANSWER CUT OFF Q)
    - Excessive (ANSWER Q ABOUT #)
    - Concomitant/Nonconcomitant
    - Medication switch
    - Inappropriate/Appropriate
    - Irrational/Rational
    - Fixed dose
    - Dual/double/Di therapy
    - Irrational
    - Other (ANSWER NEXT QUESTIONS)
  - #085(b)Other Polypharmacy clssification1 (WRITE in info box)
  - #086Other polypharmacy classification2 (WRITE in info box)
  - #087Cut off for SHORT/LONG term polypharmacy (WRITE #days in info box)
  - #088If excessive polypharmacy cut off # (WRITE in info box)
  - #089Was polypharmacy reported at class/drug level (may check >1)?
    - Not reported
    - At class level
    - At drug level
- GLOBAL PHARMACOLOGICAL CATEGORIES
  - #090Global pharmacological category reported for polypharmacy (may check >1)
    - Not reported
    - Psychotropic medications
    - Anti-infective agents
    - Analgesic, anti-inflammatory or antipyretic
    - Cardiovascular therapy agents
    - Central nervous system agents (e.g. antiepileptic)
    - Endocrine agents
    - Gastrointestinal therapy agents
    - Respiratory therapy agents
    - Supplements
    - Other (ANSWER NEXT QST)
  - #091Other global pharm cats (WRITE in info box)
- PHARMACOLOGICAL MEDICATION CATEGORIES/CLASSES
  - #092Pharmacological categories reported for polypharmacy (may check >1)
    - Not reported
    - Antianxiety
    - Antiarrhythmic
    - Antibiotic
    - Anticholinergics
    - Anticoagulant
    - Anticonvulsant
    - Antidepressant
    - Antidiarrheal
    - Antiemetic
    - Anti-flu
    - Antifungal
    - Antihistamines
    - Antihyperglycemic
    - Antimalarial
    - Antimetabolite
    - Antipsychotic
    - Antiretroviral – HIV
    - Antiretroviral - Other
    - Antitussives
    - Asthma therapy
    - ADHD meds (CNS stimulants)
    - Bipolar Therapy Agents
    - Cystic Fibrosis
    - Gastric Acid Reducers
    - Glucocorticoids
    - Hormones
    - Hypertension Therapy
    - Immunosuppressive
    - Insulins
    - Laxatives
    - Mood stabilizers
    - Non-opioids (e.g. NSAID, acetaminophen)
    - Opioids (e.g. codeine)
    - Sedatives & Hypnotics
    - Supplements
    - Other (ANSWER NEXT QST)
  - #093Other pharmacological categories (WRITE in info box)
  - #094Medication Classes included in polypharmacy (may check >1)
    - Not reported (WRITE 'none' in info box)
    - Medication class1 (WRITE in info box)
    - Medication class2 (WRITE in info box)
    - Medication class3 (WRITE in info box)
    - Medication class4 (WRITE in info box)
    - Medication class5 (WRITE in info box)
    - Medication class6 (WRITE in info box)
    - Medication class7 (WRITE in info box)
    - Medication class8 (WRITE in info box)
    - Medication class9 (WRITE in info box)
    - Medication class10 (WRITE in info box)
  - #095Was polypharmacy reported across >1 medication categories/classes?
    - NotReported
    - Yes
    - No
  - #096Medications included in polypharmacy assessment (may check >1)
    - Not reported (WRITE 'none' in info box)
    - Medication1 (WRITE in info box)
    - Medication2 (WRITE in info box)
    - Medication3 (WRITE in info box)
    - Medication4 (WRITE in info box)
    - Medication5 (WRITE in info box)
    - Medication6 WRITE in info box)
    - Medication7 (WRITE in info box)
    - Medication8 (WRITE in info box)
    - Medication9 (WRITE in info box)
    - Medication10 (WRITE in info box)
    - Medication11 (WRITE in info box)
    - Medication12 (WRITE in info box)
- DISEASES
  - #097Disease group in which polypharmacy was assessed (may check >1)
    - Not reported
    - Psychiatric disease
    - Neurological disease
    - (Other) somatic disease
    - Combination (dx grps not differentiated)
  - #098Disease in which polypharmacy was assessed (may check >1)
    - Not reported
    - ADHD
    - Adjustment disorder
    - Anxiety
    - Asthma
    - Autism
    - Bipolar disorder
    - Conduct disorder
    - Depression
    - Disruptive disorder
    - Eating/Elimination D
    - Epilepsy
    - Infections
    - Intellectual and development disorder (IDD)
    - Obsessive compulsive disorder
    - Oppositional DD
    - Other psychiatric disease
    - Other respiratory disease
    - Other somatic disease
    - Psychosis
    - PTSD
    - Substance abuse
    - Other (ANSWER NEXT QUESTIONS)
  - #099Other disease1 (WRITE in info box)
  - #100Other disease2 (WRITE in info box)
  - #101Other disease3 (WRITE in info box)
  - #102Other disease4 (WRITE in info box)
  - #103Was polypharmacy reported across >1 diseases?
    - NotReported
    - Yes
    - No
- MEDICATION HARM
  - #104What polypharmacy harms were considered (may check >1)
    - Not reported
    - Abnormal labs
    - Adverse events
    - Adverse reactions
    - Behavioral toxicities
    - Congenital abnormalities
    - Death
    - Drug-drug interactions
    - Drug resistance
    - Emergency room visit
    - Hepatotoxicity
    - High cost
    - Hospitalization
    - Impaired cognitive function
    - Irrational drug use
    - Medication error
    - Metabolic disorder
    - Nephrotoxicity
    - Non-adherence
    - Overdose
    - Pancreatitis
    - Prescription errors
    - Side effects
    - Tolerability
    - Worsening symptoms
    - Other (ANSWER NEXT QST)
  - #105Other harm1 (WRITE in info box)
  - #106Other harm2 (WRITE in info box)
  - #107Other harm3 (WRITE in info box)
- MEDICATION SAFETY
  - #108What polypharmacy benefits were considered? (may check >1)
    - Not reported
    - Adjunctive
    - Augmentation
    - Combinations for efficacy
    - Combination for safety
    - Control/treatment of complex diseases
    - De-escalation
    - Guideline based regimen
    - Medication switch
    - Potentiation/synergism
    - Rational drug use
    - Other (ANSWER NEXT QST)
  - #109Other benefit1 (WRITE in info box)
  - #110Other benefit2 (WRITE in info box)
  - #111Other benefit3 (WRITE in info box)
- CONCLUSIONS AND RECOMMENDATIONS
  - #112What were the main findings of the study?
    - Not reported (WRITE 'none')
    - Main finding1 (PASTE in info box)
    - Main finding2 (PASTE in info box)
    - Main finding3 (PASTE in info box)
  - #113Author conclusion (may check >1)
    - Not reported (WRITE 'none')
    - Conclusion1 (PASTE in info box)
    - Conclusion2 (PASTE in info box)
  - #114Author recommendation (may check >1)
    - Not reported (WRITE 'none')
    - Recommendation1 (PASTE in info box)
    - Recommendation2 (PASTE in info box)
- FINAL COMMENTS AND REVIEWER DETAILS
  - #115Confirm inclusion of study for data extraction
    - INLUDE: Not Child Stratified
    - Exclude on full text: Case report
    - Exclude on full text: Perinatal polypharmacy
    - Exclude on full text: Polypharmacy by design (RCT)
    - Exclude on full text: Not peer reviewed
    - Exclude on full text: Not children: (>=21 years)
    - Exclude on full text: Not polypharmacy
    - Exclude on full text: Not in English
    - Exclude on full text: Other reason (ANSWER NEXT QST)
    - Include on full text
  - #116Other reason for exclusion on full text (WRITE in info box)
  - #117Final comments (WRITE in info box)
  - #118Did you time the data extraction?
    - No
    - Yes
  - #119Duration of extraction (WRITE #minutes in info box)
- SIGNATURES
  - #120Primary reviewer initials
    - AH
    - CB
    - HJ
    - JS
    - JW
    - NG
    - PB
    - PK
    - RL
    - ST
    - XM
  - #121Primary review date (WRITE in info box AS mm/dd/yyyy)
  - #122Secondary reviewer initials
    - AH
    - CB
    - HJ
    - JS
    - JW
    - NG
    - PB
    - PK
    - RL
    - ST
    - XM
  - #123Secondary review date (WRITE in info box) AS mm/dd/yyyy)
  - #124Final check initials
    - PB
    - CB
    - XM
  - #125Final check date (WRITE in info box AS mm/dd/yyyy)
  - END, THANK YOU
