# Supplementary material for: A transdisciplinary team approach to scoping reviews: the case of pediatric polypharmacy
Source: BMC Med Res Methodol. 2018 Oct 4;18:102. doi: 10.1186/s12874-018-0560-4 (PMC6172739; doi:10.1186/s12874-018-0560-4)
Supplement: Supplementary file 3 — Included Studies. List of first author, year of publication, and title of all included studies. (DOCX 54 kb) [file 12874_2018_560_MOESM3_ESM.docx]

**Additional File 3: Included Studies**

| **Author** | **Title** |
| --- | --- |
| **Mental Health Conditions, Children Only Studies** | |
| Baeza (2013) | Frequency, characteristics and management of adolescent inpatient aggression |
| Baeza (2014) | Antipsychotic use in children and adolescents: a 1-year follow-up study |
| Bali (2015) | Cardiovascular Safety of Concomitant Use of Atypical Antipsychotics and Long-Acting Stimulants in Children and Adolescents With ADHD. |
| Becker (2005) | Weight changes in teens on psychotropic medication combinations at Austin State Hospital |
| Bhowmik (2013) | The utilization of psychopharmacological treatment and medication adherence among Medicaid enrolled children and adolescents with bipolar depression |
| Bhowmik (2014) | Risk of manic switch associated with antidepressant therapy in pediatric bipolar depression |
| Calarge (2012) | Correlates of weight gain during long-term risperidone treatment in children and adolescents |
| Constantine (2010) | Antipsychotic polypharmacy in the treatment of children and adolescents in the fee-for-service component of a large state Medicaid program |
| Cornblatt (2007) | Can antidepressants be used to treat the schizophrenia prodrome? Results of a prospective, naturalistic treatment study of adolescents |
| Costa (2017) | Mental health care for Brazilian juvenile offenders |
| Coury (2012) | Use of psychotropic medication in children and adolescents with autism spectrum disorders |
| Cummings (2007) | Medications prescribed for children with mood disorders: effects of a family-based psychoeducation program |
| Dean (2006) | Psychotropic medication utilization in a child and adolescent mental health service |
| Dosreis (2011) | Antipsychotic treatment among youth in foster care |
| Dusetzina (2011) | Receipt of guideline-concordant pharmacotherapy among children with new diagnoses of bipolar disorder |
| Fegert (2006) | Antidepressant use in children and adolescents in Germany |
| Fontanella (2009) | Psychotropic medication changes, polypharmacy, and the risk of early readmission in suicidal adolescent inpatients |
| Frazier (2011) | Prevalence and correlates of psychotropic medication use in adolescents with an autism spectrum disorder with and without caregiver-reported attention-deficit/hy |
| Geller (2010) | Pharmacological and non-drug treatment of child bipolar I disorder during prospective eight-year follow-up |
| Griffith (2010) | Psychotropic medication use for youth in residential treatment: A comparison between youth with monopharmacy versus polypharmacy |
| Gyllenberg (2012) | Psychotropic drug and polypharmacy use among adolescents and young adults: findings from the Finnish 1981 Nationwide Birth Cohort Study |
| Hauck (2017) | ADHD Treatment in Primary Care: Demographic Factors, Medication Trends, and Treatment Predictors |
| Hilt (2014) | Side effects from use of one or more psychiatric medications in a population-based sample of children and adolescents |
| Huefner (2017) | Patterns of psychotropic medication at admission for youth in residential care |
| Jerrell (2008) | Adverse events in children and adolescents treated with antipsychotic medications |
| Jerrell (2010) | Adverse events associated with psychotropic treatment in African American children and adolescents |
| Kamble (2015) | Concurrent Use of Stimulants and Second-Generation Antipsychotics Among Children With ADHD Enrolled in Medicaid |
| Kant (2004) | The off-label use of clozapine in adolescents with bipolar disorder, intermittent explosive disorder, or posttraumatic stress disorder |
| Korczak (2017) | Major Depressive Disorder Among Preadolescent Canadian Children: Rare Disorder or Rarely Detected? |
| Kowatch (2003) | Combination pharmacotherapy in children and adolescents with bipolar disorder |
| Kreider (2014) | Growth in the concurrent use of antipsychotics with other psychotropic medications in Medicaid-enrolled children |
| Lekhwani (2004) | Psychotropic prescription practices in child psychiatric inpatients 9 years old and younger |
| Liu (2016) | Development of a refill pattern method to measure polypharmacy in administrative claims databases. |
| Logan (2012) | High prescription drug use and associated costs among Medicaid-eligible children with autism spectrum disorders identified by a population-based surveillance net |
| Logan (2015) | Aberrant Behaviors and Co-occurring Conditions as Predictors of Psychotropic Polypharmacy among Children with Autism Spectrum Disorders |
| Madden (2017) | Psychotropic Medication Use among Insured Children with Autism Spectrum Disorder |
| Mandell (2008) | Psychotropic medication use among Medicaid-enrolled children with autism spectrum disorders |
| Masi (2005) | A Naturalistic Study of Referred Children and Adolescents With Obsessive-Compulsive Disorder |
| Masi (2009) | Pharmacotherapy in paediatric obsessive-compulsive disorder: a naturalistic, retrospective study |
| Mattison (2014) | Psychotropic medication characteristics for special education students with emotional and/or behavioral disorders |
| McIntyre (2008) | Metabolic and cardiovascular adverse events associated with antipsychotic treatment in children and adolescents |
| McIntyre (2009) | Polypharmacy in children and adolescents treated for major depressive disorder: a claims database study |
| Murray (2014) | Pharmacological treatments prescribed to people with autism spectrum disorder (ASD) in primary health care |
| Oswald (2007) | Medication use among children with autism spectrum disorders |
| Pappadopulos (2002) | "Real world" atypical antipsychotic prescribing patterns in public child and adolescent inpatient settings |
| Patel (2017) | Effect of Psychopharmacotherapy on Body Mass Index Among Children and Adolescents with Bipolar Disorders |
| Potter (2009) | Prescribing patterns for treatment of pediatric bipolar disorder in a specialty clinic |
| Raghavan (2008) | Use of multiple psychotropic medications among adolescents aging out of foster care |
| Reed (2004) | Single and combined psychotropic medication use in a child and adolescent mental health service |
| Rosenberg (2010) | Psychotropic medication use among children with autism spectrum disorders enrolled in a national registry, 2007-2008 |
| Rubin (2009) | State variation in psychotropic medication use by foster care children with autism spectrum disorder |
| Rubin (2012) | Interstate variation in trends of psychotropic medication use among Medicaid-enrolled children in foster care |
| Rushton (2001) | Pediatric stimulant and selective serotonin reuptake inhibitor prescription trends: 1992 to 1998 |
| Russell (2006) | Predictive factors for polypharmacy among child and adolescent psychiatry inpatients |
| Safer (1997) | Changing patterns of psychotropic medications prescribed by child psychiatrists in the 1990s |
| Shores (2005) | Normalization of risperidone-induced hyperprolactinemia with the addition of aripiprazole |
| Sourander (2004) | Combined psychopharmacological treatment among child and adolescent inpatients in Finland |
| Spencer (2013) | Psychotropic medication use and polypharmacy in children with autism spectrum disorders |
| Staller (2005) | Current prescribing patterns in outpatient child and adolescent psychiatric practice in central New York |
| Trinczek (2016) | Time to Initiation of Clozapine Treatment in Children and Adolescents with Early-Onset Schizophrenia |
| Wonodi (2007) | Tardive dyskinesia in children treated with atypical antipsychotic medications |
| van Wattum (2013) | Polypharmacy reduction in youth in a residential treatment center leads to positive treatment outcomes and significant cost savings |
| **Mental Health Conditions, Children and Adult Stratified Studies** | |
| Chee (2016) | Prescribing Pattern of Antidepressants in Children and Adolescents: Findings from the Research on Asia Psychotropic Prescription Pattern. |
| Essock (2009) | Identifying Clinically Questionable Psychotropic Prescribing Practices for Medicaid Recipients in New York State |
| Lake (2014) | Child, parent, and service predictors of psychotropic polypharmacy among adolescents and young adults with an autism spectrum disorder |
| Lee (2016) | Trends and intervention results for unusual antipsychotic polypharmacy prescribing patterns for Florida adult and child Medicaid population: 2007-2013: (Running |
| Morrato (2007) | Prevalence, utilization patterns, and predictors of antipsychotic polypharmacy: experience in a multistate Medicaid population, 1998-2003 |
| **Somatic Conditions, Children Only Studies** | |
| Adams (2001) | Use of inhaled anti-inflammatory medication in children with asthma in managed care settings |
| Akhtar (2012) | Drug prescribing practices in paediatric department of a north Indian university teaching hospital |
| Anghelescu (2008) | The safety of concurrent administration of opioids via epidural and intravenous routes for postoperative pain in pediatric oncology patients |
| Beksac (2016) | Postvoidal residual urine is the most significant non-invasive diagnostic test to predict the treatment outcome in children with non-neurogenic lower urinary tra |
| Blydt-Hansen (2014) | Medication treatment complexity and adherence in children with CKD |
| Brinkman (2008) | Parent-reported medication use in a head start population |
| De Souza (2016) | Off-label use and harmful potential of drugs in a NICU in Brazil: A descriptive study |
| Fadare (2015) | Drug prescribing pattern for under-fives in a paediatric clinic in South-Western Nigeria |
| Gelissen (2014) | Statin use in Australian children: a retrospective audit of four pediatric hospitals |
| Gonçalves (2015) | Drug utilisation profile in the neonatal unit of a university hospital: a prospective observational study in Brazil |
| Khoo (2000) | Drug eruptions in children: A review of 111 cases seen in a tertiary skin referral centre |
| Lass (2011) | Drug utilisation pattern and off-label use of medicines in Estonian neonatal units |
| Manjhi (2016) | Prescription pattern of antimicrobial drugs in paediatric outpatient department in a tertiary care teaching hospital |
| Najmi (2015) | Drug utilization study in the outpatient pediatric department of a tertiary care teaching hospital of district Lucknow |
| Okoro (1995) | Pattern of drug therapy in home management of diarrhoea in rural communities of Nigeria |
| Oshikoya (2006) | Evaluation of outpatient paediatric drug prescriptions in a teaching hospital in Nigeria for rational prescribing |
| Paczkowska (2016) | Compliance Among Adolescents with Arterial Hypertension. |
| Pereira (2015) | Off-label and unlicensed utilization of drugs in a Brazilian pediatric hospital. |
| Rashed (2012) | Epidemiology and potential associated risk factors of drug-related problems in hospitalised children in the United Kingdom and Saudi Arabia |
| Rizkalla (2013) | Patterns of medication exposures in hospitalized pediatric patients with acute renal failure requiring intermittent or continuous hemodialysis |
| Sarajlija (2013) | Vitamin D deficiency in Serbian patients with Rett syndrome |
| Shamshy (2011) | Drug utilization of antimicrobial drug in pediatrics population in a tertiary care hospital in erode, Tamilnadu, India |
| Sheng (2017) | Population pharmacokinetics of vancomycin in Chinese infants |
| South (2017) | Race, obesity, and the renin-angiotensin-aldosterone system: treatment response in children with primary hypertension |
| Suman (2014) | Assessment of usage of antibiotic and their pattern of antibiotic sensitivity test among childhood fever |
| Ungar (2008) | Effect of cost-sharing on use of asthma medication in children |
| Vishwanath (2014) | Assessment of drug utilization in hospitalized children at a tertiary care teaching hospital |
| Yoon (2012) | Antihypertensive prescribing patterns for adolescents with primary hypertension |
| Zeng (2017) | A prospective study of the use of antibiotics in the Emergency Department of a Chinese University Hospital |
| Zimmerman (1987) | Prevalence of abnormalities found by sinus x-rays in childhood asthma: lack of relation to severity of asthma |
| de las (2016) | Adverse drug reactions in hospitalized Colombian children |
| **Somatic Conditions, Children and Adult Stratified Studies** | |
| Dodoo (2009) | Pattern of drug utilization for treatment of uncomplicated malaria in urban Ghana following national treatment policy change to artemisinin-combination therapy |
| Njoku (2017) | Lack of benefit with combination therapy for clostridium difficile infection |
| **Epilepsy, Children Only Studies** | |
| Adewuya (2005) | Prevalence of and risk factors for anxiety and depressive disorders in Nigerian adolescents with epilepsy |
| Agbato (1986) | Total and free serum concentrations of carbamazepine and carbamazepine-10,11-epoxide in children with epilepsy |
| Al-Qudah (1991) | Contribution of carbamazepine-10,11-epoxide to neurotoxicity in epileptic children on polytherapy |
| Albsoul-Younes (2016) | Patterns of antiepileptic drugs use in epileptic pediatric patients in Jordan. |
| Aldenkamp (2005) | Educational underachievement in children with epilepsy: a model to predict the effects of epilepsy on educational achievement |
| Allarakhia (1996) | Valproic acid and thrombocytopenia in children: a case-controlled retrospective study |
| Anderson (2015) | A prospective study of adverse drug reactions to antiepileptic drugs in children. |
| Arteaga (1993) | PLATELET GABA-TRANSAMINASE IN EPILEPTIC CHILDREN - INFLUENCE OF EPILEPSY AND ANTICONVULSANTS |
| Asadi-Pooya (2005) | Drug compliance of children and adolescents with epilepsy |
| Atugonza (2016) | Multiple anti-epileptic drug use in children with epilepsy in Mulago hospital, Uganda: A cross sectional study |
| Bansal (2017) | Predictors of health related quality of life in childhood epilepsy and comparison with healthy children: findings from an Indian study |
| Bassili (2002) | Pattern of diagnostic of childhood epilepsy and therapeutic care in Alexandria, Egypt |
| Batista (2007) | Evaluation of sleep habits in children with epilepsy |
| Beckung (1997) | Hidden dysfunction in childhood epilepsy |
| Belanger (1998) | Role of vigabatrin and lamotrigine in treatment of childhood epileptic syndromes |
| Braakman (2012) | Cognitive and behavioural findings in children with frontal lobe epilepsy |
| Carpay (1998) | Epilepsy in childhood: an audit of clinical practice |
| Cepelak (1998) | Valproate and carbamazepine comedication changes hepatic enzyme activities in sera of epileptic children |
| Chakova (1998) | Effect of antiepileptic drugs on the thyroid gland in children with epilepsy (preliminary report) |
| Chakraborty (2013) | Study of behavioral and cognitive disorders in school going children with epilepsy |
| Chan (1997) | Determination of phenobarbitone population clearance values for physically and mentally handicapped Chinese children with epilepsy |
| Chen (2007) | Reinvestigation and reduction of polytherapy in children with chronic seizures |
| Chiba (1985) | Comparison of steady-state pharmacokinetics of valproic acid in children between monotherapy and multiple antiepileptic drug treatment |
| Cho (2015) | Trends in Antiepileptic Drug Prescriptions for Childhood Epilepsy at a Tertiary Children's Hospital in Korea, 2001-2012. |
| Choudhary (2014) | Behavioral comorbidity in children and adolescents with epilepsy |
| Chung (1997) | Alterations in the carnitine metabolism in epileptic children treated with valproic acid |
| Cloyd (1993) | Valproic acid pharmacokinetics in children. IV. Effects of age and antiepileptic drugs on protein binding and intrinsic clearance |
| Coppola (1997) | Lamotrigine as add-on drug in children and adolescents with refractory epilepsy and mental delay: An open trial |
| Coppola (2012) | Role of folic acid depletion on homocysteine serum level in children and adolescents with epilepsy and different MTHFR C677T genotypes |
| Covanis (1992) | Absence epilepsy: early prognostic signs |
| Cull (1992) | CHANGES IN ANTIEPILEPTIC DRUG REGIMEN AND BEHAVIOR IN CHILDREN WITH EPILEPSY |
| Czochanska (1996) | Intractable epilepsy in children who develop epilepsy in the first decade of life--a prospective study |
| Desai (2011) | Does One More Medication Help? Effect of Adding Another Anticonvulsant in Childhood Epilepsy |
| El-Khayat (2003) | Physical and hormonal profile of male sexual development in epilepsy |
| El-Rashidy (2015) | Cardiac autonomic balance in children with epilepsy: value of antiepileptic drugs |
| Eldeen (2012) | Homocysteine, folic acid and vitamin B12 levels in serum of epileptic children |
| Ferreira (2001) | Childhood epilepsy due to neurocysticercosis: a comparative study |
| Fong (2016) | Vitamin D deficiency and its risk factors in Malaysian children with epilepsy. |
| Freilinger (2006) | Behavioral and emotional problems in children with epilepsy |
| Furlanut (1985) | Carbamazepine and carbamazepine-10,11-epoxide serum concentrations in epileptic children |
| Galas-Zgorzalewicz (1996) | The effect of chronic carbamazepine, valproic acid and phenytoin medication on the periodontal condition of epileptic children and adolescents |
| Gebauer-Bukurov (2015) | Social competence among well-functioning adolescents with epilepsy |
| Gray (1998) | A model for the determination of carbamazepine clearance in children on mono- and polytherapy |
| Hallioglu (2008) | Effects of antiepileptic drug therapy on heart rate variability in children with epilepsy |
| Hasan (2010) | Antiepileptic drug utilisation and seizure outcome among paediatric patients in a Malaysian public hospital |
| Hassanien (2010) | Tissue plasminogen activator in children with idiopathic and intractable epilepsies |
| Helal (2013) | Monotherapy versus polytherapy in epileptic adolescents |
| Hernandez (2002) | Deficits in executive functions and motor coordination in children with frontal lobe epilepsy |
| Heyman (2012) | Lamotrigine serum concentration in children with epilepsy |
| Hoie (2005) | Seizure-related factors and non-verbal intelligence in children with epilepsy. A population-based study from Western Norway |
| Ikawa (1999) | Influence of concomitant anticonvulsants on serum concentrations of clonazepam in epileptic subjects: An age- and dose-effect linear regression model analysis |
| Incecik (2014) | Risk of recurrence after discontinuation of antiepileptic drug therapy in children with epilepsy |
| Jozwiak (2011) | Antiepileptic treatment before the onset of seizures reduces epilepsy severity and risk of mental retardation in infants with tuberous sclerosis complex |
| Kanemura (2015) | The Relationship between Nocturnal Enuresis and Sequential Changes of NAG/Morning Urine Gravity in Epileptic Children Treated with Valproate Sodium |
| Kanta (2014) | Common prescribing errors in childhood epilepsy: A report from a tertiary care teaching hospital of Northern India |
| KarasalIhoGlu (2003) | Risk factors of status epilepticus in children |
| Kim (2009) | Hypohidrosis related symptoms in pediatric epileptic patients with topiramate |
| Ko (2001) | Valproic acid and thrombocytopenia: cross-sectional study |
| Korinthenberg (1994) | The metabolization of carbamazepine to CBZ-10,11-epoxide in children from the newborn age to adolescence |
| Kothare (2004) | Efficacy and tolerability of zonisamide in juvenile myoclonic epilepsy |
| Kwon (2004) | The potential for QT prolongation by antiepileptic drugs in children |
| Kwong (1998) | Epilepsy in children with cerebral palsy |
| Kwong (2016) | Anxiety and Depression in Adolescents With Epilepsy. |
| Lagunju (2016) | Seizure-related injuries in children and adolescents with epilepsy. |
| Lai (2015) | Validation of the Neuro-QoL measurement system in children with epilepsy |
| Larson (2012) | Impact of pediatric epilepsy on sleep patterns and behaviors in children and parents |
| Lee (2015) | Longitudinal change of vitamin D status in children with epilepsy on antiepileptic drugs: prevalence and risk factors |
| Liu (1994) | Improved therapeutic monitoring of drug interactions in epileptic children using carbamazepine polytherapy |
| Liu (2015) | Influence of valproic acid concentration and polymorphism of UGT1A4*3, UGT2B7 -161C>T and UGT2B7*2 on serum concentration of lamotrigine in Chinese epileptic chi |
| Masri (2014) | Manifestations and treatment of epilepsy in children with neurometabolic disorders: a series from Jordan |
| Mathew (2012) | An initial experience with therapeutic drug monitoring of levetiracetam as reported from a pediatric clinical setting in India |
| Matricardi (2016) | Epilepsy in the setting of full trisomy 18: A multicenter study on 18 affected children with and without structural brain abnormalities. |
| Matricardi (2016) | Neuropsychological profiles and outcomes in children with new onset frontal lobe epilepsy. |
| Mert (2011) | Factors affecting epilepsy development and epilepsy prognosis in cerebral palsy |
| Mikati (2007) | Risk factors for development of subclinical hypothyroidism during valproic acid therapy |
| Nakajima (2011) | Evaluation of valproate effects on acylcarnitine in epileptic children by LC-MS/MS |
| Nettekoven (2008) | Effects of antiepileptic drug therapy on vitamin D status and biochemical markers of bone turnover in children with epilepsy |
| Nolan (2003) | Intelligence in childhood epilepsy syndromes |
| Novak (1997) | Risk factors for status epilepticus in children with symptomatic epilepsy |
| Novak (2005) | Acute drug prescribing to children on chronic antiepilepsy therapy and the potential for adverse drug interactions in primary care |
| O'Leary (2006) | Performance of children with epilepsy and normal age-matched controls on the WISC-III |
| Oguz (2002) | Relationship of epilepsy-related factors to anxiety and depression scores in epileptic children |
| Ohtsuka (1988) | Rational treatment of refractory epilepsy in childhood |
| Ohtsuka (2000) | Refractory childhood epilepsy and factors related to refractoriness |
| Opala (1991) | The effect of valproic acid on plasma carnitine levels |
| Osama (2016) | Social skills among epileptic adolescents |
| Otsuka (1994) | Urinary N-acetyl-beta-glucosaminidase and guanidinoacetic acid levels in epileptic patients treated with anti-epileptic drugs |
| Poudel (2016) | Predictors of Poor Seizure Control in Children Managed at a Tertiary Care Hospital of Eastern Nepal. |
| Racaru (2013) | Sleep architecture impairment in epileptic children and putative role of anti epileptic drugs |
| Raty (2003) | Seizures and therapy in adolescents with uncomplicated epilepsy |
| Reilly (2014) | Academic achievement in school-aged children with 'active' epilepsy: A population-based study |
| Reilly (2014) | Neurobehavioral comorbidities in children with active epilepsy: a population-based study |
| Riechmann (2015) | Costs of epilepsy and cost-driving factors in children, adolescents, and their caregivers in Germany |
| Roeder (2009) | Depression and mental health help-seeking behaviors in a predominantly African American population of children and adolescents with epilepsy |
| Rufo-Campos (2006) | Long-term use of oxcarbazepine oral suspension in childhood epilepsy: open-label study |
| Rytter (2009) | Antiepileptic drug treatment of children at a referral centre for epilepsy--does admission make a difference? |
| Ryu (2015) | Perceived stigma in Korean adolescents with epilepsy: Effects of knowledge about epilepsy and maternal perception of stigma |
| Rzezak (2007) | Frontal lobe dysfunction in children with temporal lobe epilepsy |
| Rzezak (2011) | Episodic and semantic memory in children with mesial temporal sclerosis |
| Salih (2012) | Characteristics of seizure frequency among Malaysian children diagnosed with structural-metabolic epilepsy |
| Salih (2012) | Medical care costs of newly diagnosed children with structural-metabolic epilepsy: a one year prevalence-based approached |
| Schwabe (2001) | Clinical experience with topiramate dosing and serum levels in children 12 years or under with epilepsy |
| Selassie (2008) | Speech, language, and cognition in preschool children with epilepsy |
| Sheth (2007) | Bone mineral density with lamotrigine monotherapy for epilepsy |
| Smith (2002) | Cognitive skills in children with intractable epilepsy: comparison of surgical and nonsurgical candidates |
| Sobaniec (1992) | Certain aspects of interaction between sodium valproate and other anticonvulsant drugs in the therapy of epilepsy in children |
| Sobaniec (2006) | Evaluation of the influence of antiepileptic therapy on antioxidant enzyme activity and lipid peroxidation in erythrocytes of children with epilepsy |
| Star (2014) | Valproic acid and fatalities in children: a review of individual case safety reports in VigiBase |
| Sugimoto (1996) | Valproate metabolites in high-dose valproate plus phenytoin therapy |
| Summers (1986) | The effect of a specialist clinic with pharmacist involvement on the management of epilepsy in paediatric patients |
| Suzuki (1991) | Valproic acid dosages necessary to maintain therapeutic concentrations in children |
| Talarska (2011) | Cognitive functioning and behaviour of epileptic children in parents' assessment |
| Tekgul (2005) | Antiepileptic drug-induced osteopenia in ambulatory epileptic children receiving a standard vitamin D3 supplement |
| Thome-Souza (2003) | Lamotrigine and valproate: efficacy of co-administration in a pediatric population |
| Turkdogan (2002) | Lipid peroxidation and antioxidative enzyme activities in childhood epilepsy |
| Turkdogan (2003) | Visual and auditory event related potentials in epileptic children: a comparison with normal and abnormal MRI findings |
| Udani (1993) | Difficult to control epilepsy in childhood--a long term study of 123 cases |
| Uldall (1999) | Clinical experiences with topiramate in children with intractable epilepsy |
| Unay (2006) | Evaluation of renal tubular function in children taking anti-epileptic treatment |
| Valencia (2005) | Efficacy and tolerability of topiramate in children younger than 2 years old |
| Van De Vrie-Hoekstra (2008) | Antiepileptic drug utilization in children from 1997-2005 - A study from the Netherlands |
| Verrotti (1999) | Carnitine deficiency and hyperammonemia in children receiving valproic acid with and without other anticonvulsant drugs |
| Verrotti (2004) | Factors associated with poor control in partial complex epilepsy |
| Verrotti (2012) | Antiepileptic drug withdrawal in childhood epilepsy: what are the risk factors associated with seizure relapse? |
| Wang (1993) | Clinical significance of measurement of % free valproic acid in epileptic children |
| Wang (2016) | Utilization of antiepileptic drugs on monotherapy and polytherapy for children at Shanghai in China |
| Williams (1996) | The effects of seizure type, level of seizure control, and antiepileptic drugs on memory and attention skills in children with epilepsy |
| Williams (2003) | Anxiety in children with epilepsy |
| Yukawa (2001) | Influence of age and comedication on steady-state clonazepam serum level-dose ratios in Japanese epileptic patients |
| Zubcevic (2010) | Outcome of patients with infantile spasms |
| **Epilepsy, Children and Adult Stratified Studies** | |
| Alexandre (2010) | Characteristics of a large population of patients with refractory epilepsy attending tertiary referral centers in Italy |
| Armijo (1997) | Vigabatrin serum concentration to dosage ratio: influence of age and associated antiepileptic drugs |
| Battino (1980) | Carbamazepine plasma levels in children and adults: Influence of age, dose, and associated therapy |
| Bryant (1996) | Valproic acid hepatic fatalities. III. U.S. experience since 1986 |
| Cramer (2011) | Non-interventional surveillance study of adverse events in patients with epilepsy |
| Di Rosa (2013) | Role of plasma homocysteine levels and MTHFR polymorphisms on IQ scores in children and young adults with epilepsy treated with antiepileptic drugs |
| El-Hajj (2008) | Predictors of bone density in ambulatory patients on antiepileptic drugs |
| Farhat (2002) | Effect of antiepileptic drugs on bone density in ambulatory patients |
| Franco (2014) | Off-label prescribing of antiepileptic drugs in pharmacoresistant epilepsy: a cross-sectional drug utilization study of tertiary care centers in Italy |
| Hamer (2012) | Prevalence, utilization, and costs of antiepileptic drugs for epilepsy in Germany - A nationwide population-based study in children and adults |
| Javed (2011) | Local epidemiological survey of epilepsy |
| Junger (2015) | The Pediatric Epilepsy Side Effects Questionnaire: Establishing clinically meaningful change |
| Kadam (2014) | Retrospective study on therapeutic drug monitoring of lamotrigine in Indian epileptic patients |
| Kondo (1992) | Associations between risk factors for valproate hepatotoxicity and altered valproate metabolism |
| Ohtahara (2004) | Safety of zonisamide therapy: prospective follow-up survey.[Republished in Seizure. 2007 Jan;16(1):87-93; PMID: 17269155] |
| Sanchez (1986) | Steady-state carbamazepine plasma concentration-dose ratios in epileptic patients |
| Yamamoto (2014) | Impact of cytochrome P450 inducers with or without inhibitors on the serum clobazam level in patients with antiepileptic polypharmacy |
| Yukawa (1991) | Influence of age and concurrent medication on steady-state valproic acid serum level-dose ratios in Japanese paediatric patients |
| Yukawa (1992) | Influence of age and co-medication on steady-state carbamazepine serum level-dose ratios in Japanese paediatric patients |
| Yukawa (1992) | Influence of age and co-medication on steady-state phenobarbital serum level-dose ratios in Japanese paediatric patients |
| **Combined disease Conditions, Children Only Studies** | |
| Abdel-Salam (2000) | Association of epilepsy with different groups of microcephaly |
| Amitai (2015) | Effects of long-term valproic acid treatment on hematological and biochemical parameters in adolescent psychiatric inpatients: A retrospective naturalistic study |
| Aras (2007) | Medication prescribing practices in a child and adolescent psychiatry outpatient clinic |
| Betts (2014) | Period Prevalence of Concomitant Psychotropic Medication Usage Among Children and Adolescents with Attention-Deficit/Hyperactivity Disorder During 2009 |
| Birarra (2017) | Assessment of drug-related problems in pediatric ward of Zewditu Memorial Referral Hospital, Addis Ababa, Ethiopia |
| Blader (2006) | Pharmacotherapy and postdischarge outcomes of child inpatients admitted for aggressive behavior |
| Brauner (2016) | Off-Label Prescription of Psychopharmacological Drugs in Child and Adolescent Psychiatry |
| Comer (2010) | National trends in child and adolescent psychotropic polypharmacy in office-based practice, 1996-2007 |
| Connor (1997) | Combined pharmacotherapy in children and adolescents in a residential treatment center |
| Dafoulis (2012) | Factors associated with behavioral problems in children with idiopathic epilepsy |
| Duffy (2005) | Concomitant pharmacotherapy among youths treated in routine psychiatric practice |
| Fontanella (2014) | Trends in psychotropic polypharmacy among youths enrolled in Ohio Medicaid, 2002-2008 |
| Ghose (1983) | Hypercupraemia induced by antiepileptic drugs |
| Gururaj (2003) | Epilepsy in children with cerebral palsy |
| Hadjipanayis (1997) | Epilepsy in patients with cerebral palsy |
| Hobbs (2014) | Clinicians' utilization of child mental health telephone consultation in primary care: findings from Massachusetts |
| Hong (2010) | Anticholinergic use in children and adolescents after initiation of antipsychotic therapy |
| Jain (2013) | Obstructive sleep apnea and primary snoring in children with epilepsy |
| Jameel (2012) | A descriptive study of use of psychotropic drugs in child and adolescent psychiatric illness in an inpatient facility |
| Kaplan (2016) | Anticonvulsant Efficacy in Sturge-Weber Syndrome |
| Kelly (2004) | Atypical Antipsychotic Use in a State Hospital Inpatient Adolescent Population |
| Knezevic-Pogancev (2011) | Epileptic sezure reapearance risk, afther antiepileptic drug withdrawal in children with cerebral palsy |
| Kowatch (2013) | Prescription of psychiatric medications and polypharmacy in the LAMS cohort |
| Kulak (2003) | Risk factors and prognosis of epilepsy in children with cerebral palsy in north-eastern Poland |
| Liu (1994) | The influence of polytherapy on the relationships between serum carbamazepine and its metabolites in epileptic children |
| Marchand (2004) | Quetiapine Adjunctive and Monotherapy for Pediatric Bipolar Disorder: A Retrospective Chart Review |
| Martin (2003) | Multiple psychotropic pharmacotherapy among child and adolescent enrollees in Connecticut Medicaid managed care |
| Olashore (2017) | Polypharmacy among children and adolescents with psychiatric disorders in a mental referral hospital in Botswana |
| Olashore (2017) | Diagnostic profiles and predictors of treatment outcome among children and adolescents attending a national psychiatric hospital in Botswana |
| Olashore (2017) | Prescribing pattern of psychotropic medications in child psychiatric practice in a mental referral hospital in Botswana |
| Osunsanmi (2016) | Influence of Age, Gender, and Living Circumstances on Patterns of Attention-Deficit/Hyperactivity Disorder Medication Use in Children and Adolescents With or Wit |
| Patel (2017) | Care Provision and Prescribing Practices of Physicians Treating Children and Adolescents With ADHD |
| Procyshyn (2014) | Prevalence and patterns of antipsychotic use in youth at the time of admission and discharge from an inpatient psychiatric facility |
| Radigan (2005) | Medication patterns for attention-deficit/hyperactivity disorder and comorbid psychiatric conditions in a low-income population |
| Reilly (2015) | Cognition in school-aged children with "active" epilepsy: A population-based study |
| Saldana (2014) | Antipsychotic polypharmacy in children and adolescents at discharge from psychiatric hospitalization |
| Schubart (2014) | Psychotropic medication trends among children and adolescents with autism spectrum disorder in the Medicaid program |
| Singhi (2003) | Epilepsy in children with cerebral palsy |
| Sullivan (2015) | Psychopharmacological treatment among adolescents with disabilities: Prevalence and predictors in a nationally representative sample. |
| Thome-Souza (2004) | Which factors may play a pivotal role on determining the type of psychiatric disorder in children and adolescents with epilepsy? |
| Valente (2013) | Angelman syndrome caused by deletion: a genotype-phenotype correlation determined by breakpoint |
| Weng (2016) | Increased risks of tic disorders in children with epilepsy: A nation-wide population-based case-control study in Taiwan |
| Zito (2008) | Psychotropic medication patterns among youth in foster care |
| dos (2006) | Adverse drug reactions in hospitalized children in Fortaleza, Brazil |
| dosReis (2005) | Multiple psychotropic medication use for youths: a two-state comparison |
| **Combined disease Conditions, Children and Adult Stratified Studies** | |
| Dreifuss (1987) | Valproic acid hepatic fatalities: a retrospective review |
| Dreifuss (1989) | Valproic acid hepatic fatalities. II. US experience since 1984 |
| Esbensen (2009) | A longitudinal investigation of psychotropic and non-psychotropic medication use among adolescents and adults with autism spectrum disorders |
| Kalilani (2017) | Lacosamide and concomitant use of antiepileptic and other medications in a US population — A retrospective cohort study |
| Landmark (2007) | Clinical use of antiepileptic drugs at a referral centre for epilepsy |
| Malerba (2010) | Patterns of prescription of antiepileptic drugs in patients with refractory epilepsy at tertiary referral centres in Italy |
| Sadahiro (1985) | Effect of serum albumin on free fractions of phenobarbital and valproic acid in patients with convulsive seizures |
| Sarkar (2013) | Pattern of psychotropic prescription in a tertiary care center: a critical analysis |
| **Disease Condition Not Specified, Children Only Studies** | |
| Albatti (2017) | The self-medication use among adolescents aged between 13–18 years old; Prevalence and behavior, Riyadh – Kingdom of Saudi Arabia, from 2014–2015 |
| Allaire (2016) | Morbid obesity and use of second generation antipsychotics among adolescents in foster care: Evidence from Medicaid |
| Bellis (2014) | Adverse drug reactions and off-label and unlicensed medicines in children: a prospective cohort study of unplanned admissions to a paediatric hospital |
| Bhatara (2004) | National trends in concomitant psychotropic medication with stimulants in pediatric visits: practice versus knowledge |
| Brenner (2014) | Use of Psychotropic Medications among Youth in Treatment Foster Care |
| Burch (2011) | Using a Trigger Tool to Assess Adverse Drug Events in a Children's Rehabilitation Hospital |
| Chatterjee (2007) | Drug utilization study in a neonatology unit of a tertiary care hospital in eastern India |
| Chen (2011) | The definition and prevalence of pediatric psychotropic polypharmacy |
| Choure (2017) | Drug utilization study in neonatal intensive care unit at rural tertiary care hospital |
| Dai (2016) | Epidemiology of Polypharmacy and Potential Drug-Drug Interactions Among Pediatric Patients in ICUs of U.S. Children's Hospitals. |
| Daniell (1989) | Audit of drug usage in a regional neonatal intensive care unit |
| Dash (2015) | Monitoring of adverse drug reactions in pediatric department of a Tertiary Care Teaching Hospital: A hospital based observational study |
| Dedefo (2016) | Incidence and determinants of medication errors and adverse drug events among hospitalized children in West Ethiopia. |
| Du (2006) | Changing patterns of drug utilization in a neonatal intensive care population |
| Faber (2005) | Psychotropic co-medication among stimulant-treated children in the Netherlands |
| Fattahi (2005) | Adverse drug reactions in hospitalized children in a department of infectious diseases |
| Feinstein (2015) | The depth, duration, and degree of outpatient pediatric polypharmacy in Colorado fee-for-service Medicaid patients. |
| Feinstein (2015) | Potential drug-drug interactions in infant, child, and adolescent patients in children's hospitals |
| Feudtner (2011) | Pediatric palliative care patients: A prospective multicenter cohort study |
| Feudtner (2012) | Prevalence of polypharmacy exposure among hospitalized children in the United States |
| Getachew (2016) | Potential drug-drug interactions in pediatric wards of Gondar University Hospital, Ethiopia: A cross sectional study |
| Gilat (2011) | Trends in prescribing of psychotropic medications for inpatient adolescents in Israel: a 10 years retrospective analysis |
| Hug (1991) | Reduction of serum carnitine concentrations during anticonvulsant therapy with phenobarbital, valproic acid, phenytoin, and carbamazepine in children |
| Isenalumhe (1988) | Polypharmacy: its cost burden and barrier to medical care in a drug-oriented health care system |
| Jurgens (2014) | Hospital readmission in children with complex chronic conditions discharged from subacute care |
| Khan (2013) | Adverse drug reactions in hospitalized pediatric patients of Saudi Arabian University Hospital and impact of pharmacovigilance in reporting ADR |
| Kieran (2014) | Unlicensed and off-label drug use in an Irish neonatal intensive care unit: A prospective cohort study |
| Knopf (2010) | Perceived adverse drug reactions among non-institutionalized children and adolescents in Germany |
| Kovess (2015) | Psychotropic Medication Use in French Children and Adolescents |
| Kumar (2008) | Medication Use in the Neonatal Intensive Care Unit: Current Patterns and Off-Label Use of Parenteral Medications |
| Kurian (2016) | Adverse Drug Reactions in Hospitalized Pediatric Patients: A Prospective Observational Study |
| Lemer (2009) | The role of advice in medication administration errors in the pediatric ambulatory setting |
| Mancini (2006) | Anxiolytics, hypnotics, and antidepressants dispensed to adolescents in a French region in 2002 |
| Martinbiancho (2007) | Profile of drug interactions in hospitalized children |
| Mattison (1999) | Use of psychotropic medications in special education students with serious emotional disturbance |
| Nduka (2017) | Prescribing practices for pediatric out-patients: A case study of two teaching hospitals in Nigeria |
| Neubert (2010) | Drug utilisation on a preterm and neonatal intensive care unit in Germany: A prospective, cohort-based analysis |
| Ng (2002) | Safety of benzodiazepines in newborns |
| Olfson (2002) | National trends in the use of psychotropic medications by children |
| Palikhe (2004) | Prescribing pattern of antibiotics in paediatric hospital of Kathmandu valley |
| Pandey (2010) | Prescription analysis of pediatric outpatient practice in nagpur city |
| Pise (2015) | Drug prescribing and dispensing pattern in pediatrics outpatient clinic of a rural tertiary-care teaching hospital |
| Rashed (2012) | Risk factors associated with adverse drug reactions in hospitalised children: international multicentre study |
| Rashed (2014) | Epidemiology and potential risk factors of drug-related problems in Hong Kong paediatric wards |
| Sharma (2016) | Antibiotic prescribing patterns in the pediatric emergency department at Georgetown Public Hospital Corporation: a retrospective chart review. |
| Thrane (1999) | Multiple prescriptions of antibiotics for children aged 0 to 5 years in relation to type of antibiotic |
| Vernacchio (2009) | Medication use among children <12 years of age in the United States: results from the Slone Survey |
| Wammanda (2003) | Drug treatment costs: Projected impact of using the integrated management of childhood illnesses |
| Warrier (2006) | Patterns of drug utilization in a neonatal intensive care unit |
| Zandieh (2008) | Risk factors in preventable adverse drug events in pediatric outpatients |
| Zoega (2009) | Psychotropic drug use among Icelandic children: a nationwide population-based study |
| **Disease Condition Not Specified, Children and Adult Stratified Studies** | |
| Apanga (2014) | Evaluation of drug prescribing pattern under the national health insurance scheme in rural Ghana |
| Becker (2013) | Antipsychotic polypharmacy prescribing patterns and costs in the Florida adult and child Medicaid populations |
| Bergendal (2015) | Concomitant use of two or more antipsychotic drugs is common in Sweden |
| Datta (2016) | Patterns of prescription and antibiotic use among outpatients in a tertiary care teaching hospital of Bangladesh |
| Hincapie-Castillo (2017) | Prevalence of Psychotherapy Surrounding Initiation of Psychotropic Polypharmacy in the Medicaid-Insured Population, 1999-2010 |
| Hovstadius (2009) | Dispensed drugs and multiple medications in the Swedish population: an individual-based register study |
| Hovstadius (2010) | Increasing polypharmacy - an individual-based study of the Swedish population 2005-2008 |
| Rotermann (2014) | Prescription medication use by Canadians aged 6 to 79 |
| Vallano (2004) | Medical specialty and pattern of medicines prescription |
