# Supplementary material for: A transdisciplinary team approach to scoping reviews: the case of pediatric polypharmacy
Source: BMC Med Res Methodol. 2018 Oct 4;18:102. doi: 10.1186/s12874-018-0560-4 (PMC6172739; doi:10.1186/s12874-018-0560-4)
Supplement: Supplementary file 4 — Inter-rater Concordance Measures for Screening on Title & Abstract, and Full Text. Details of percent agreement and kappa statistics for screening title & abstracts as well as full texts. (DOCX 19 kb) [file 12874_2018_560_MOESM4_ESM.docx]

**Additional File 4: Inter-rater Concordance Measures for Screening on Title & Abstract, and Full Text**

| **Phase (Number)** | **Include/Exclude** | | **Include/Exclude with Reason** | |
| --- | --- | --- | --- | --- |
|  | **Percent Agreement** | **Kappa (95% CI)** | **Percent Agreement** | **Kappa (95% CI)** |
| **Screening on Title and Abstract (25% inclusion proportion)** | | | | |
| Implementation, Tertile 1 (377) | 74.0 | 0.44 (0.35-0.53)^a^ | 46.7 | 0.32 (0.27-0.38)^a^ |
| Implementation, Tertile 2 (368) | 82.3 | 0.55 (0.45-0.64)^a^ | 64.6 | 0.56 (0.50-0.63)^a^ |
| Implementation, Tertile 3 (368) | 86.1 | 0.66 (0.57-0.74)^a^ | 76.4 | 0.70 (0.64-0.76)^a^ |
| Update (158) | 82.3 | 0.57 (0.43-0.71)^a^ | 65.6 | 0.56 (0.46-0.65)^a^ |
| Hand Search (327) | 73.1 | 0.42 (0.32-0.52)^a^ | 65.7 | 0.53 (0.46-0.61)^a^ |
| **Overall (1,598)** | **80.2** | **0.54 (0.50-0.59)^a^** | **65.8** | **0.57 (0.54-0.60)^a^** |
| **Screening on Full Text (30% inclusion proportion)** | | | | |
| Implementation, Quintile 1 (172) | 81.4 | 0.58 (0.45-0.71)^a^ | 70.4 | 0.61 (0.52-0.70)^a^ |
| Implementation, Quintile 2 (171) | 76.0 | 0.50 (0.38-0.63)^a^ | 64.3 | 0.54 (0.45-0.63)^a^ |
| Implementation, Quintile 3 (173) | 89.0 | 0.75 (0.64-0.85)^a^ | 82.1 | 0.76 (0.68-0.83)^a^ |
| Implementation, Quintile 4 (161) | 86.3 | 0.71 (0.60-0.83)^a^ | 85.1 | 0.80 (0.73-0.87)^a^ |
| Implementation, Quintile 5 (169) | 91.1 | 0.79 (0.69-0.89)^a^ | 84.6 | 0.80 (0.73-0.87)^a^ |
| Update (71) | 88.7 | 0.77 (0.62-0.92)^a^ | 83.1 | 0.76 (0.64-0.88)^a^ |
| Hand Search (93) | 89.3 | 0.79 (0.66-0.91)^a^ | 82.8 | 0.76 (0.65-0.86)^a^ |
| **Overall (1,010)** | **85.5** | **0.69 (0.64-0.73)^a^** | **78.1** | **0.71 (0.68-0.74)^a^** |
| **Total (2,608)** | **82.3** | **0.60 (0.57-0.63)^a^** | **70.7** | **0.67 (0.65-0.69)^a^** |

^a^ p-value <.001; CI=Confidence Interval
